# The Use of Granulocyte Colony-Stimulating Factor as Primary Prophylaxis in Patients Undergoing Chemotherapy: An Experience of Moroccan Oncologists

**DOI:** 10.7759/cureus.54482

**Published:** 2024-02-19

**Authors:** Mohamed Kaakoua, Soukayna Boujmadi, Rhizlane Belbaraka, Ismail Essadi

**Affiliations:** 1 Medical Oncology Department, Ibn Sina Military Hospital, Marrakesh, MAR; 2 Department of Medical Oncology, Mohammed VI University Hospital Center, Marrakesh, MAR

**Keywords:** primary prophylaxis, morocco, chemotherapy, g-csf, febrile neutropenia, neutropenia

## Abstract

Febrile neutropenia (FN) is a frequent and serious emergency for oncologic patients undergoing chemotherapy. Using granulocyte colony-stimulating factor (G-CSF) as primary prophylaxis of febrile neutropenia is an integral part of the management of cancer patients. Our study aims to identify the challenges that prevent Moroccan oncologists from prescribing G-CSF for primary prevention.

Seventy doctors participated in our study, with a participation rate of 35.35%. Twenty-two participants had at least five years of experience in oncology. Most participants were medical oncologists (82.9%), and two-thirds of them practiced in teaching hospitals. Regarding the use of G-CSF in primary prevention, all participants complied with the recommendations for FN risk assessment and the prescription of G-CSF for prophylaxis in patients at high risk of FN (>20%). However, their use in intermediate-risk patients remains limited mainly by the cost of these drugs (45.7% of participants).

FN remains a dreadful complication in oncology. Since the introduction of G-CSF into standard oncology practice, particularly in primary prevention, the management of certain patients has improved considerably. Nevertheless, the indications for G-CSF in our context, essentially in intermediate-risk patients, are uncertain.

## Introduction

Febrile neutropenia (FN) is a medical emergency in oncology that can be life-threatening, mainly in patients undergoing chemotherapy. Studies suggest that febrile neutropenia (FN) occurs in approximately 10% to 15% of patients with solid tumors, and the mortality rate associated with it ranges from 4% to 12.5% [1,2]. There are many risk factors for febrile neutropenia, including those related to the cancer disease (tumour stage and bone marrow invasion), those related to treatment (treatment protocol and dose), and those related to the patient (age, general condition, co-morbidity) [3]. The use of granulocyte colony growth factors (G-CSF) as primary prophylaxis in patients undergoing conventional chemotherapy has clearly improved the risk of occurrence of febrile neutropenia and its morbidity and mortality [4]. G-CSF has also made it possible to maintain anticancer treatment, sometimes even at high doses (dose intensity) [5]. The European Organisation for the Research and Treatment of Cancer (EORTC) group has classified chemotherapy protocols according to the risk of occurrence of febrile neutropenia into three groups: a high-risk group (risk of FN more than 20%), an intermediate-risk group (between 15-20%) and the low-risk group (<10%) [6]. Several scientific societies (European Society for Medical Oncology, National Comprehensive Cancer Network, and EORTC) recommend G-CSF as primary prophylaxis for high-risk treatments of FN. However, their place in intermediate-risk protocols is unclear [6-8]. The purpose of our work is to evaluate the use and prescription of growth factors (G-CSF) by Moroccan oncologists as primary prophylaxis in cancer patients undergoing chemotherapy.

## Materials and methods

Study design and population

This is an expert-based survey study carried out among doctors from Moroccan cancer centers (public, private, and military). A study spread over a period of six months, from November 2022 to May 2023. We included all cancerologists (medical oncologists and radiotherapists) registered on the list of physician members of the Moroccan Cancer Society (SMC) and practicing in Moroccan cancer centers (public, private, and military). This includes physicians at all levels (resident, specialist, and professor).

Data collection

We collected data by sending an electronic questionnaire via e-mail (using the Google Forms app) to all physicians who are members of the Moroccan Cancer Society. The questionnaire begins with the consent of the participants before answering the questionnaire. Afterward, we analyzed the participant's responses anonymously.

We divided our questionnaire into two distinct parts. The first part focuses on the analysis of the characteristics of the participants (the participant's status, experience, speciality, sector, and city of practice), while the second part reports on their practices regarding the use of G-CSF for primary prophylaxis. In particular, the risk factors for neutropenia, cancers, and therapeutic classes causing the occurrence of neutropenia, the main issues limiting the use of G-CSF in primary prophylaxis, And its impact on the prognosis of cancer disease.

Ethical consideration

For this study, the research ethics committee did not need to approve it, as only the participants who answered the questionnaire accepted and approved their consent. However, we requested authorization by email from the ethics committee of the Moroccan Cancer Society.

Data analysis

Statistical analysis was carried out using IBM SPSS statistics software, version 24.0. To present quantitative variables, we utilized mean, while for qualitative variables, we displayed them using percentages and numbers.

## Results

Part one: a descriptive study

We sent our questionnaire to 198 doctors who are members of the Moroccan Cancer Society and have their e-mail addresses registered. Only 70 of them responded to the questionnaire, i.e. a participation rate of 35.35%. Females dominate our population, with 45 women (64.3%) and 25 men (35.7%), a female-to-male sex ratio of 1.8.

In our series (n: 70), the age groups were 30 to 39 years old and less than 29 years old, representing 48.6% (n: 34) and 24.3% (n: 17) of all participants, respectively. Of all participants, 15 individuals (21.4%) were 40 to 49 years old, and only four individuals (5.7%) were above 50 years old. More than half of the participants (n: 41) had less than 5 years of experience in oncology (58.6%). Participants with more than 10 years of experience accounted for 30% (n: 21), and only 11.4% (n: 8) had between five and 10 years of experience.

**Figure 1:**
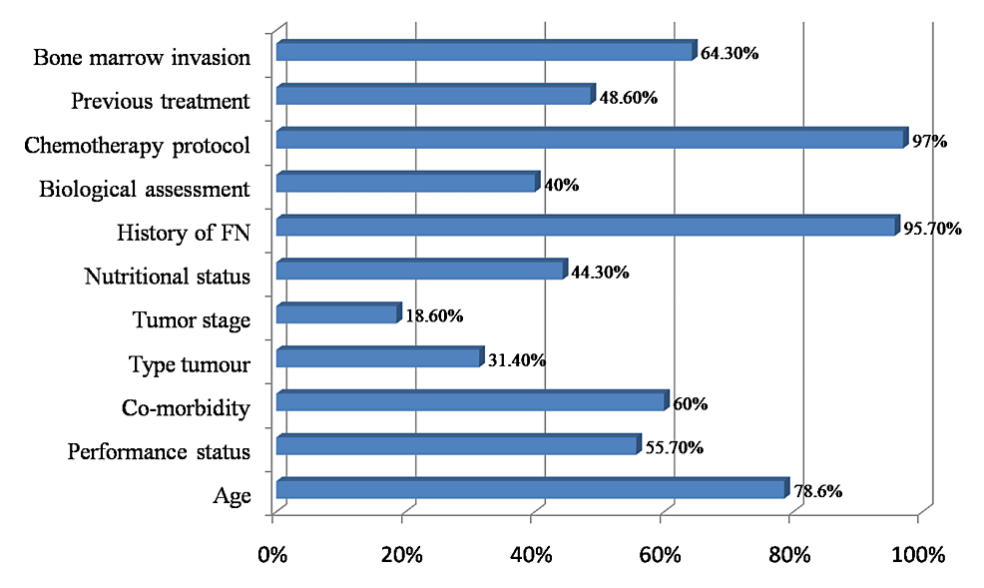
Risk factors to take into consideration when assessing the risk of neutropenia (n: 70)

The majority of participants (n: 58) were medical oncologists (82.9%), and only 17.1% of participants were radiation therapists (n: 12) (Figure 1). Almost half of the participants (n: 37) were resident doctors (52.9%). While specialists and professors represented 31.4% (n: 22) and 15.7% (n: 11) of participants, respectively. Table 1 shows the different participant demographics.

**Table 1:** The different participant demographics

Variables		Number	Percentage (%)
Gender	Women	45	35.70%
	Men	25	64.30%
Age range	< 29 years	17	24.30%
	30–39 years	34	48.60%
	40–49 years	15	21.40%
	> 50 years	4	5.70%
Oncology experience	< 5 years	41	58.60%
	5–10 years	8	11.40%
	> 10 years	21	30%
Participant speciality	Medical oncologist	58	82.90%
	Radiation therapist	12	17.10%
Professional status	Resident doctor	37	52.90%
	Specialist	22	31.40%
	Professor	11	15.70%

Almost 2/3 of the participants (n: 45) work in teaching hospitals (64.3%). Doctors in private cancer centers and military hospitals represented 18.6% and 14.3% of participants, respectively. Only 2.9% of participants work in public centers. The distribution of participating doctors according to their place of practice was as follows: 40% of participants practise in Marrakesh (28 doctors), 21.4% in Tangier (15 doctors), 15.7% in Rabat (11 doctors) and 12.9% in Casablanca (9 doctors). Three doctors practise in Agadir (4.3%), 2 in Fez (2.8%) and only 1 doctor in Meknes and Elhoceima (1.4%).

Part two: analysis of current practice

In this part, we asked participants for their views on the risk factors favoring the occurrence of FN episodes in patients undergoing chemotherapy, the factors justifying the prescription of G-CSF as primary prophylaxis and the limiting factors of their prescription.

The vast majority of participants considered that the chemotherapy protocol (97.1%; n: 68) and the history of FN (95.7%; n: 67) were the major factors to be taken into account when assessing the risk of neutropenia. More than half the participants reported age (78.6%; n: 55), bone marrow invasion (64.3%; n: 45), co-morbidities (60%; n: 42) and the patient's general condition (55.7%; n: 39). Tumour type and stage were reported in 31.1% (n: 21) and 18.6% (n: 13) respectively (Figure 1).

**Figure 2:**
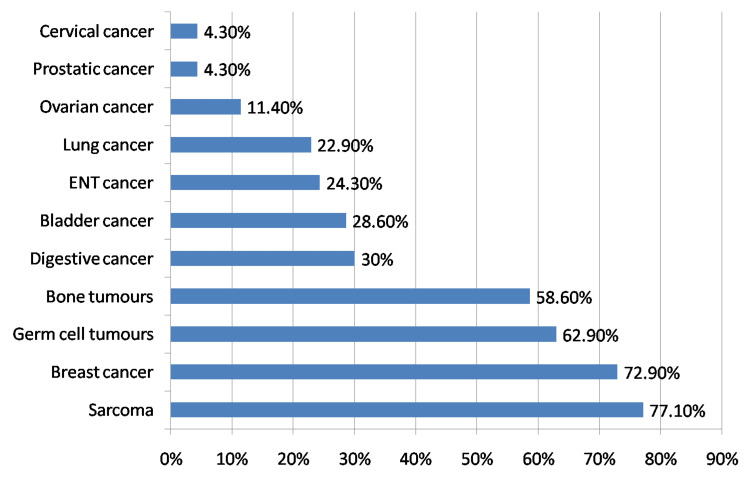
The use of G-CSF as primary prophylaxis according to the type of cancer G-CSF: Granulocyte colony-stimulating factor

Most participants stated that the chemotherapy protocol (88.6%; n: 62), the dose-intensity regimen (88.6%; n: 62), and the history of FN (82.9%; n: 58) were the determining factors for using G-CSF as primary prophylaxis. For a minority of participants, this prophylaxis also depended on the following factors: initial biological tests (28.6%; n: 20), previous chemotherapy lines (28.6%; n: 20), number of chemotherapy sessions received (18.6%; n: 13) and tumor type (11.4%; n: 8). Concerning, drugs most likely to cause neutropenia and justifying the prescription of G-CSF as primary prophylaxis are anthracyclines (78.6%; n: 55), followed by alkylating agents (57.1%; n: 40), taxanes (41.4%; n: 29), platinum salts (41.4%; n: 29) and antimetabolites (27.1%; n: 19). According to the participant's experience, the tumours most often requiring primary prophylaxis with G-CSF are: sarcomas (77.1%; n: 54), breast cancer (72.9%; n: 51), germ cell tumours (62.9%; n: 44) and bone tumours (58.6%; n: 41). However, cervical cancer (4.3%; n: 3) and prostate cancer (4.3%; n: 3) are the sites least in need of primary prophylaxis (Figure 2).

**Figure 3:**
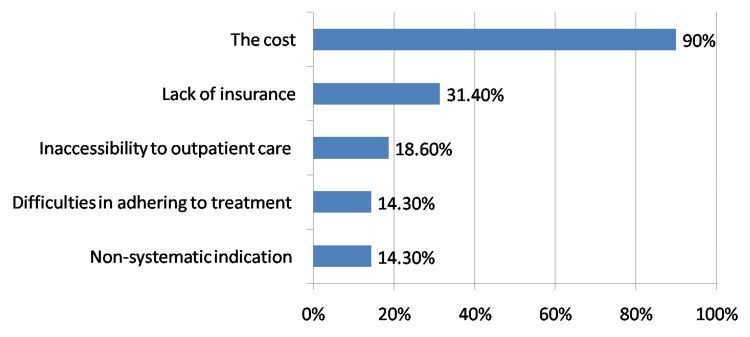
The main issues limiting the prescription of G-CSF G-CSF: Granulocyte colony-stimulating factor

Regarding the molecules used and methods of prescribing G-CSF for primary prophylaxis. The majority of participants used to prescribe filgrastim for prophylaxis (95.7%; n: 67), while only nine participants (12.9%; n: 9) used pegfilgrastim and four (5.7%) others used lenograstim. Half the participants prescribed G-CSF within 24 hours of the chemotherapy treatment, a quarter waited 48 hours before prescribing it, and only 14.3% (n: 10) used it after 72 hours. Two-thirds of participants used an average of five injections of G-CSF (excluding the pegylated form), while 15.7% (n: 11) used three injections and 7.1% recommended four. According to participants, the main obstacle to the use of growth factors in primary prophylaxis remains the cost associated with these drugs (90% of participants; n: 63). The second challenge is the lack of insurance coverage for these drugs (31.4%; n: 22). Finally, patient's inaccessibility to outpatient care (18.6%; n: 13), difficulties in adhering to treatment (14.3%; n: 10) and non-systematic indication (14.3%; n: 10) are minor issues (Figure 3).

Nearly half of the participants (n: 33) prescribed G-CSF for situations at high risk of febrile neutropenia and sometimes even those at intermediate risk, while the other half prescribed it only for situations at high risk. Almost all participants (98.6%; n: 69) felt that the use of G-CSF as primary prophylaxis has an impact on the prognosis of cancer disease.

## Discussion

Despite therapeutic progress and development in oncology, chemotherapy still has a place in certain types of cancer. Unlike targeted therapies, everyone widely knows that conventional chemotherapy causes myelosuppression effects, such as anemia, neutropenia, and thrombocytopenia. Chemotherapy-induced neutropenia remains a frequent and serious toxicity because of its complications: infectious, which can be life-threatening and oncological related to cessation or delay of the administration of chemotherapy.

Granulocyte colony-stimulating factors (G-CSF) are an integral part of the prevention (primary and secondary) and management of febrile neutropenia. The use of G-CSF accelerates neutrophil maturation by stimulating their granulocyte progenitors and reduces the duration and depth of neutropenia [9].

Assessing the risk of neutropenia in patients undergoing chemotherapy appears to be crucial before each cycle. The EORTC has described several factors (linked to the patient, the treatment, and the cancerous disease) to help assess this risk [6]. These factors include age over 65, general condition of the patient, stage of the disease, a history of febrile neutropenia, chemotherapy protocol, difficulties in accessing G-CSF, and absence of antibiotic prophylaxis [6]. The participants in our study apply these different factors in their practices to assess the risk of neutropenia when managing their patients.

The EORTC has classified chemotherapy protocols into 3 levels according to the risk of occurrence of febrile neutropenia. We distinguish between high-risk protocols (risk >20%), intermediate-risk protocols (10-20%), and low-risk protocols (<10%) [6]. However, several studies have shown that chemotherapy based on platinum salts, anthracyclines or taxanes was associated with an increased rate of febrile neutropenia [10-12]. Our study's results align perfectly with these findings.

The incidence of FN in patients with solid tumors undergoing chemotherapy is lower than in patients treated for hematologic malignancies [13, 14]. However, mortality secondary to this complication is contrastingly higher in patients with solid tumors (10.1% versus 7.3%) [15]. The frequent use of G-CSF in the treatment of hematologic malignancies partly contributes to this difference. The study identified bronchial cancer as the solid tumor with the highest mortality rate from chemo-induced FN, followed by gastric cancer and breast cancer [15]. In our series, the participants prefer to use growth factors mainly in the management of sarcomas, breast cancer, and germ cell tumors.

In the absence of other equally effective and less toxic chemotherapy options, healthcare professionals strongly recommend G-CSFs as the primary prevention for chemotherapy protocols at high risk of FN. They are not necessary for patients with low risk, while the recommendations for intermediate-risk are not very clear and must take into account factors relating to the patient, the cancer, and its treatment [6-8]. In our series, all participants agreed with the use of G-CSF as primary prophylaxis for high-risk situations (>20%). However, half the patients (45.7%) also preferred to use them occasionally for intermediate-risk situations.

In Morocco, there are three G-CSF molecules (lenograstim, filgrastim, and pegfilgrastim). All three drugs can be administered by intravenous infusion or subcutaneous injection. Only pegfilgrastim can be used as a single injection; the other two molecules are used according to the chemotherapy protocol on a daily basis so that they cover the nadir of neutropenia [16,17]. In our series, more than 95% of participants prescribed filgrastim, essentially due to its cost, which was the main limiting factor for prescribing G-CSF for primary prevention in 90% of participants.

Several studies and meta-analyses have reported on the benefits and impact of the use of G-CSF prophylaxis on the prognosis and survival of patients undergoing chemotherapy. In addition to reducing the risk of FN and mortality linked to serious infections, G-CSF significantly reduces the need to decrease chemotherapy doses and consequently improves adherence to cancer treatment. This benefit also has a socio-economic dimension, manifested by a reduction in hospital days and the use of antibiotics [18]. In our study, almost all participants (98.6%) admitted the positive impact of G-CSF on patient survival.

Much will undoubtedly continue to be written about the role of G-CSF in oncology, particularly in patients undergoing chemo-immunotherapy. Several preclinical studies have shown the modulatory effects of G-CSF on lymphocytes, which may reduce the efficacy of immunotherapy [19].

Limitations of our study

The low participation rate (35.35%) in our study will not allow us to draw conclusions on current clinical practices in primary prophylaxis of NF with G-CSF in cancer centers in Morocco (the place of G-CSF in our practices, the modalities of use of G-CSF, the real challenges limiting their prescription). Also, because of the heterogeneity of the participants (public, private, and military sectors) and the need to respect the anonymity of the participants, it was not possible to determine the use of G-CSF according to the sector of practice in order to confirm whether the place of practice has an impact on prescribing.

Recommendation

In Morocco, where access to supportive care is not yet generalized among the entire population, healthcare providers must incorporate the use of G-CSF in chemotherapy protocols at high risk of febrile neutropenia. However, oncologists should consider several factors (socio-economic, intellectual, etc.) of the patient to adapt the use of G-CSF in intermediate-risk situations and avoid hospitalizations and additional management of febrile neutropenia.

The Moroccan health system needs to understand the financial challenges of caring for patients with febrile neutropenia, such as hospitalization and medical tests. We should implement practical strategies to reduce costs and improve access to G-CSF for chemotherapy patients.

## Conclusions

FN remains a serious complication in oncology. In Morocco, the incorporation of G-CSF into chemotherapy protocols is a necessity for chemotherapy protocols with high risk and intermediate risk of febrile neutropenia. The cost of treatment remains the crucial factor limiting their use in primary prevention, which undoubtedly needs to be overcome in order to improve the care of our patients.
